# Nutraceutical or Pharmacological Potential of *Moringa oleifera* Lam.

**DOI:** 10.3390/nu10030343

**Published:** 2018-03-12

**Authors:** Xianjuan Kou, Biao Li, Julia B. Olayanju, Justin M. Drake, Ning Chen

**Affiliations:** 1 Tianjiu Research and Development Center for Exercise Nutrition and Foods, Hubei Key Laboratory of Exercise Training and Monitoring, College of Health Science, Wuhan Sports University, Wuhan 430079, China; kouxianjuan@126.com (X.K.); 17375002427@163.com (B.L.); 2 Rutgers Cancer Institute of New Jersey, New Brunswick, NJ 08901, USA; olayanmo@scarletmail.rutgers.edu; 3 Department of Medicine, Division of Medical Oncology, Rutgers Robert Wood Johnson Medical School, New Brunswick, NJ 08901, USA; 4 Department of Pharmacology, Rutgers Robert Wood Johnson Medical School, New Brunswick, NJ 08901, USA

**Keywords:** *Moringa oleifera*, pharmacological potential, chronic disease, flavonoids, cancer, metabolism

## Abstract

*Moringa oleifera* Lam. (*M. oleifera*), which belongs to the Moringaceae family, is a perennial deciduous tropical tree, and native to the south of the Himalayan Mountains in northern India. *M. oleifera* is rich in proteins, vitamin A, minerals, essential amino acids, antioxidants, and flavonoids, as well as isothiocyanates. The extracts from *M. oleifera* exhibit multiple nutraceutical or pharmacological functions including anti-inflammatory, antioxidant, anti-cancer, hepatoprotective, neuroprotective, hypoglycemic, and blood lipid-reducing functions. The beneficial functions of *M. oleifera* are strongly associated with its phytochemicals such as flavonoids or isothiocyanates with bioactivity. In this review, we summarize the research progress related to the bioactivity and pharmacological mechanisms of *M. oleifera* in the prevention and treatment of a series of chronic diseases—including inflammatory diseases, neuro-dysfunctional diseases, diabetes, and cancers—which will provide a reference for its potential application in the prevention and treatment of chronic diseases or health promotion.

## 1. Introduction

*Moringa oleifera* Lam. *(M. oleifera*) is a cruciferous plant that belongs to the Moringaceae family. *M. oleifera* is commonly called horseradish tree or drumstick tree by locals and is a popular staple in different parts of the world. *M. oleifera* is consumed not only for its nutritional values but also its medical benefits [1]. *M. oleifera* leaves are rich in beta-carotene, vitamin C, vitamin E, and polyphenols and are a good source of natural antioxidants [2]. Currently, *M. oleifera* is reported to enhance a broad range of biological functions including anti-inflammatory, anti-cancer, hepatoprotective, and neuroprotective functions [1,3,4]. In addition, many studies have revealed its therapeutic value including anti-diabetes, anti-rheumatoid arthritis, anti-atherosclerosis, anti-infertility, pain relief, anti-depression, and diuretic and thyroid regulation [5,6]. Due to these reported functions, the bioactivity of *M. oleifera* has gained tremendous attention over the last decade, thereby leading to the increasing exploration and understanding of its pharmacological functions and underlying mechanisms. In this review, we summarize current research progress related to its nutraceutical or pharmacological functions and corresponding mechanism of action, as well as potential benefits for human health. 

## 2. Antimicrobial Activity

A series of investigations have been conducted to evaluate the antimicrobial activity of *Moringa* species with the reports that the extracts from different parts of the *M. oleifera* plant—including seeds, stem bark, leaves, and root bark—can exert antimicrobial potential [7,8,9,10,11,12]. For example, the water-soluble lectin isolated from the extract of *M. oleifera* seeds has inhibitory effects on the growth, survival, and cell permeability of multiple species of pathological bacteria [9]. In addition, the extract of *M. oleifera* roots are reported to contain an active antibiotic pterygospermin that has powerful antibacterial and fungicidal effects [12]. The aglycone of deoxy-niazimicine isolated from the chloroform fraction of an ethanol extract of *M. oleifera* root bark is found to be responsible for antibacterial and antifungal activities [10], while the juice from the stem bark exhibits an antibacterial effect against *Staphylococcus aureus* [8]. The aqueous and ethanolic extracts from the leaves of *M. oleifera* have promising anti-bacterial properties, with strong inhibitory effects on Gram-positive species (*Staphylococcus aureus* and *Enterococcus faecalis*) over Gram-negative species (*Escherichia coli*, *Salmonella*, *Pseudomonas aeruginosa*, *Vibrio parahaemolyticus*, and *Aeromonas caviae*) [11]. In addition, the ethanol extract from leaves of *M. oleifera* has demonstrated the highest mean inhibitory zone against the growth of both *S. aureus* and *Streptococcus* mutants during the comparison between experimental toothpaste containing the extract from different parts of the *M. oleifera* plant versus mouthwash solutions [10].

## 3. Anti-Inflammation

Inflammation is a physiological response to protect the body against infection and restore tissue injury [13]. However, long-term chronic inflammation may lead to the development of chronic inflammation-associated diseases and disorders such as diabetes, cancer, autoimmune diseases, cardiovascular diseases, sepsis, colitis, and arthritis [14,15]. Inflammatory cytokines such as interleukin-1 beta (IL-1β) and tumor necrosis factor alpha (TNF-α) can upregulate the production of nitric oxide (NO) and prostaglandin E2 (PGE-2), thus stimulating the expression or enhancing the activity of inducible NO synthase (iNOS), cyclooxygenase-2 (COX-2), and microsomal PGE synthase-1 (mPGES-1) in target cells [16]. *M. oleifera* has been reported to not only decrease the production of TNF-α, IL-6, and IL-8 in response to both lipopolysaccharide (LPS) and cigarette smoke extract (CSE)-stimulated human monocyte-derived macrophages (MDM), but also inhibit the expression of RelA, a gene in nuclear factor-kappa B (NF-κB) p65 signaling during inflammation [17]. Moreover, in acetic acid-induced acute colitis rat models, oral administration of hydro-alcoholic extract from *M. oleifera* seeds (MSHE) at three increasing doses (50, 100, and 200 mg/kg) can reduce distal colon weight as a marker of inflammation and tissue edema, ulcer and mucosal inflammation severity, crypt damage, invasion involvement, total colitis index, and myeloperoxidase (MPO) activity when compared with the untreated groups [18]. So it can be considered as an alternative remedy for inflammatory bowel disease (IBD) and/or the preventive strategy of its recurrence in acetic acid-induced acute colitis rat models. Furthermore, previous studies have documented that *M. oleifera* can selectively inhibit the production of iNOS and COX-2 and significantly inhibit the secretion of NO and other inflammatory markers—including PGE-2, TNF-α, IL-6, and IL-1β—in lipopolysaccharide (LPS)-induced RAW264.7 cells. Meanwhile, it can induce the production of IL-10 in LPS-stimulated macrophages in a dose-dependent manner, thereby contributing to the suppression of NF-κB signaling pathway [19,20]. The novel bioactive phenolic glycosides 4-[(2-*O*-acetyl-α-l-rhamnosyloxy)benzyl] isothiocyanate (RBITC) from *M. oleifera* inhibited expression of COX-2 and iNOS at both the protein and mRNA levels through inhibiting major upstream signaling pathways mitogen-activated protein kinases (MAPKs) and NF-κB [21]. In vivo, an isothiocyanate-enriched *M. oleifera* seed extract (MSE) has shown a reduction in carrageenan-induced rat paw edema, which is comparable to aspirin. In vitro, its major isothiocyanate (MIC-1) at the dose of 5 μM can significantly reduce inflammatory cytokines. Further, MIC-1 at a dose of 10 μM can also have stronger effects, when compared to curcumin, on upregulating nuclear factor (erythroid-derived 2)-like 2 (Nrf2) target genes NAD(P)H: quinone oxidoreductase 1 (NQO1), glutathione S-transferase pi 1 (GSTP1), and heme oxygenase 1 (HO-1) [22]. 

**Figure 1:**
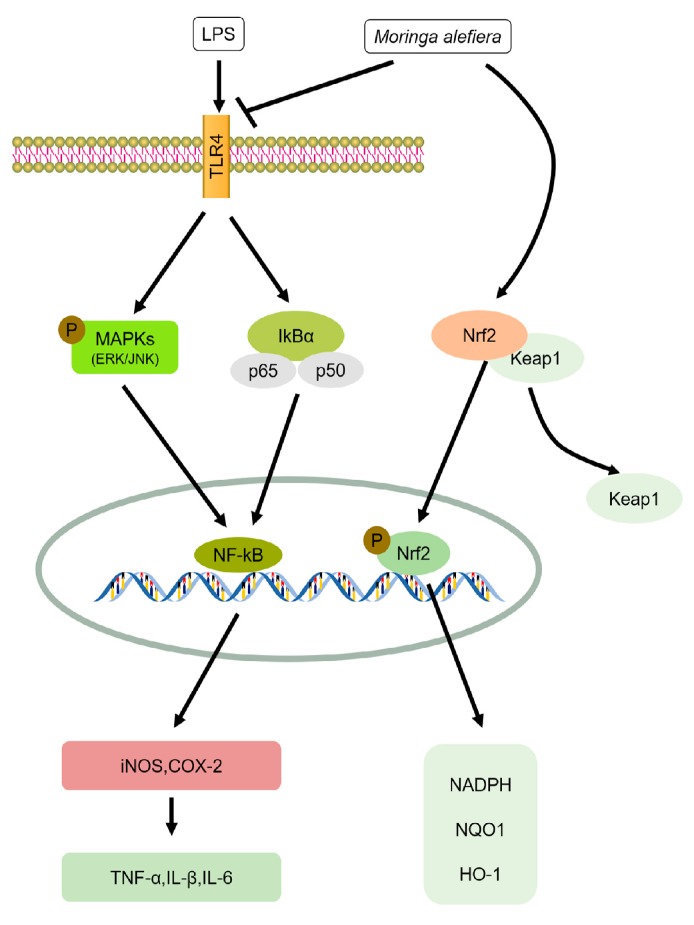
The anti-inflammatory mechanisms of *M. oleifera.* Schematic diagram illustrating the signaling pathways involved in the inhibitory effect of *M. oleifera* on proteins associated with LPS-induced inflammation summarized from a series of previous studies [17,19,20,21,22]. Toll-like receptor 4, TLR4; Nicotinamide adenine dinucleotide phosphate, NADPH; Inhibitor of kappa B, IκB; Kelch-like erythroid cell-derived protein with cap’n’collar (CNC) homology (ECH)-associated protein 1, KEAP1. Lipopolysaccharide, LPS; mitogen-activated protein kinases, MAPKs; c-Jun N-terminal kinase, p-JNK; extracellular signal-related kinase, ERK; nuclear factor (erythroid-derived 2)-like 2, Nrf2; nuclear factor-kappa B, NF-κB; inducible NO synthase: iNOS; cyclooxygenase-2, COX-2; tumor necrosis factor alpha, TNF-α; interleukin-1 beta, IL-β; interleukin-6, IL-6; quinone oxidoreductase 1, NQO1; heme oxygenase 1, HO-1.

Finally, in a clinical study of 15 patients with urinary tract infection, Maurya and Singh observed that 66.67% of patients were completely cured of their symptoms after a three-week treatment with *M. oleifera* bark extract, while 13.33% reported moderate relief from their symptoms, 13.33% of patients had no symptom change, and 6.67% relapsed in the trial group. However, in the control group, 46.67% of patients were cured, 26.66% of patients were relieved from their symptoms, 6.67% of patients had no symptom change, and 20% relapsed [23]. This study suggests *M. oleifera* bark extract is effective on most of the cardinal symptoms of urinary tract infection. These findings further support the traditional application of *M. oleifera* as an effective treatment for inflammation. The corresponding molecular mechanisms are summarized in Figure 1.

## 4. Antioxidant and Hepatoprotective Effects

Usually, natural compounds rich in polyphenols have strong antioxidant properties and can decrease oxidative damage in tissues by scavenging free radical [24,25,26]. The methanol extract of *M. oleifera* leaves contains chlorogenic acid, rutin, quercetin glucoside, and kaempferol rhamnoglucoside, whereas in the root and stem barks, several procyanidin peaks are detected [27]. Similarly, the *Moringa* genus has high antioxidant activity mainly due to its high content of bioactive polyphenols [28,29]. Fortunately, as a medicinal plant, *M. oleifera* extracts from both mature and tender leaves exhibit strong antioxidant activity against free radicals and prevent oxidative damage due to the enrichment of polyphenols [29].

Lipid peroxidation (LPO) plays an important role in the metabolism of the body, which can lead to cell lesion and nerve damage if internal and external balances are broken. In a radiation-induced Swiss albino mouse model with oxidative stress, the pre-treatment with *M. oleifera* leaf extract for 15 consecutive days can effectively restore glutathione (GSH) level and prevent lipid peroxidation in liver [30,31]. This protective effect may be related to a variety of phytochemicals such as ascorbic acid and phenols (catechin, epicatechin, ferulic acid, ellagic acid, and myricetin) through scavenging radiation-induced free radicals. Moreover, in an acute paracetamol (PCM)-induced hepatotoxicity model, the pre-administration of the hydro-ethanolic extract of *M. oleifera* before oral administration of PCM at the dose of 3 g/kg to male Sprague Dawley rats results in a significant reduction of lipid peroxidation; interestingly, the levels of glutathione-S transferase (GST), glutathione peroxidase (GPx), and glutathione reductase (GR) are restored to the normal levels in the group subjected to the pre-administration of *M. oleifera* extract [32]. These results are equivalent to the positive control silymarin (200 mg/kg; p.o.) and exhibits similar results to other research teams [33,34]. Furthermore, daily oral post-treatment with *M. oleifera* leaf extract (100, 200, and 400 mg/kg body weight) of the rats with carbon tetrachloride (CCl_4_)-induced lipid peroxidation and hepatic damage for 60 consecutive days can protect CCl_4_-induced hepatotoxicity, which may be due to the presence of total phenols and flavonoids in the extract and/or the purified compounds such as β-sitosterol, quercetin and kaempferol [35]. Similarly, previous finding has also demonstrated that the post-treatment of *M. oleifera* leaf extract for consecutive 28 days can protect from cadmium-induced hepatotoxicity of the rats through suppressing the elevated alkaline phosphatase (ALP), glutamic oxaloacetic transaminase (aspartate aminotransferase, AST), glutamic pyruvic transaminase (alanine aminotransferase, ALT), and LPO levels and increasing superoxide dismutase (SOD) level [36]. Furthermore, the oral administration of *M. oleifera* extract also reveals a significant protective action to the liver damage induced by anti-tubercular drug such as isoniazid (INH), rifampicin (RMP), or pyrazinamide (PZA), as evidenced by the recovered AST, ALT, ALP, and bilirubin levels in serum, as well as the reduced lipid peroxidation in liver [36]. The extract of *M. oleifera* leaves can also effectively reduce high-fat-diet (HFD)-induced liver damage of mice [37]. Compared with the model group, the treatment with the leaf extract of *M. oleifera* protects HFD-induced liver damage as indicated by recusing the abnormal histopathological change and AST, ALT, and ALP activity, and stimulates a significant increase in endogenous antioxidant parameters [37]. Overall, these data suggest that the extract of *M. oleifera* has both preventive and curative functions for liver tissue.

## 5. Neuroprotective Effect

Dementia—a serious loss of global cognitive capacity including impaired memory, attention, language, and problem-resolving capacity—is a progressive neurodegenerative disorder that is growing worldwide due to an increased aging population [38]. Alzheimer’s disease (AD) is the most common cause of dementia that is an irretrievable chronic neurodegenerative disease. ROS-associated with oxidative stress can induce cell apoptosis through mitochondrial dysfunction, and result in the damage of lipids, proteins and DNA [39,40]. Previous studies have shown that oxidative stress is believed to be a primary factor in neurodegenerative diseases including AD, Parkinson’s disease (PD) and Huntington’s disease (HD), as well as amyotrophic lateral sclerosis (ALS) [41]. Therefore, antioxidants have gained extensive attention as promising therapeutic agents for neurodegenerative diseases. Although many efforts in the discovery of new treatments for AD have been uncovered, none of the existing treatments have been shown to slow or halt the progression of this disease [42]. Due to the high cost of synthetic anti-dementia drugs and corresponding side effects, natural products containing flavonoids have gained tremendous interest as candidates for the prevention and/or treatment of neurodegenerative disorders [39,42]. The extract from the leaves of *M. oleifera* are thought to exhibit both antioxidant activity and nootropic effects. Indeed, the alcoholic extract of *M. oleifera* leaves can combat oxidative stress in a rat model with AD induced by colchicines [43]. In 1-methyl-4-phenyl-1,2,3,6-tetrahydropyridine (MPTP)-induced sub-acute PD mouse model, the pre-treatment with isothiocyanate isolated from the extract of *M. oleifera* seeds for one week not only modulated the signal pathway for inflammation, but also regulated the signaling pathways associated with oxidative stress and apoptosis. The efficacy of *M. oleifera* in countering inflammatory signal pathway has been corroborated by in vitro results, which can be used in clinical practice as a useful drug for the prevention or treatment of PD [44].

*M. oleifera* has been shown to stimulate neuronal outgrowth and survival under harsh treatment conditions [45,46]. For example, a concentration of 30 μg/mL ethanol extract from the leaves of *M. oleifera* can promote the outgrowth of neurites and neuronal differentiation from primary embryonic neurons in a concentration-dependent manner [45]. Similarly, *M. oleifera* leaf extract has been observed to increase the number and length of dendrites and axonal branches, the length of axons, and eventually facilitate synaptogenesis [45]. Previous studies have also demonstrated that *M. oleifera* leaf extract can successfully improve spatial memory and neurodegeneration in cornu ammonis 1 (CA1), CA2, and CA3 regions, and dentate gyrus of hippocampal tissues [46]. Mechanically, it can also decrease malondialdehyde (MDA) levels and acetylcholinesterase (AChE) activity, but can increase SOD and catalase (CAT) activity. In addition, compared with the aluminum-alone group, the administration of *M. oleifera* leaf extract at the dose of 300 mg/kg for 28 consecutive days in rats with aluminum chloride-induced temporal cortical degeneration protected against aluminum chloride-induced neurotoxicity of the temporal cortex of rats by decreasing the expression of neuron specific enolase (NSE) and glial fibrillary acidic protein (GFAP) [47].

As more people struggle with depression, a serious health problem in most countries, the need for efficient intervention or treatment options is paramount. Because of the side effects of anti-depressants during long-term application, the discovery of safer anti-depressant herbal remedies is necessary. *M. oleifera* is a potential remedy for treating nervous system disorders acting as a memory-enhancing agent. A previous study [48] in standardized mouse models with depression confirmed that the anti-depressant effect of the alcoholic extract from *M. oleifera* leaves may be invoked through the noradrenergic-serotonergic neurotransmission pathway after administrating *M. oleifera* extract at the daily dose of 200 mg/kg coupled with fluoxetine at the daily dose of 10 mg/kg for 14 consecutive days. This suggests that the combinatorial administration of *M. oleifera* and fluoxetine or other selective serotonin reuptake inhibitor (SSRI) drugs seems to have promising potential.

## 6. Anticancer Property of *M. oleifera*

Cancer is the second leading cause of death in the United States and a prominent cause of death worldwide [49]. Effective therapeutic approaches have been adopted to treat various types of cancers, however, resistance and/or toxicity creates the need for more effective treatment options. 

Several epidemiological studies have established a negative correlation between consumption of cruciferous vegetables and risk of breast, lung, and colon cancer [50,51]. *M. oleifera* leaf and bark extracts have been shown to effectively inhibit the growth of breast, pancreatic, and colorectal cancer cells [52,53]. Gas chromatography-mass spectroscopy (GC-MS) analysis by Alsamari and colleagues documented 12 different compounds in *M. oleifera* extract, 3 of which may have anticancer properties [52]. Isothiocyanates, which have been described as a potent anticancer compound, occur naturally in its precursor form, glucosinolates, in an intact plant. Glucosinolates are hydrolyzed in a reaction catalyzed by the enzyme myrosinase to produce isothiocyanate when the intact plant is disrupted [54]. 

Isothiocyanates have been extensively studied for their anticancer properties. Xiao et al. have reported that allyl isothiocyanates (AITC) inhibits the growth of androgen independent (PC-3) and androgen dependent (LNCaP) human prostate cancer cells [55]. This study also established a correlation between the inhibition of growth of PC-3 cells in the presence of AITC and gap2/mitosis (G2/M) cell accumulation coupled with apoptosis. Reduction in the protein levels of cyclin-dependent kinase 1 (CDK1), cell division cycle protein 25B (CDC25B), and CDC25C was observed after treating PC-3 and LNCaP cells with AITC for 24 h. Boreddy and colleagues treated mice with BxPC-3 tumor xenografts with benzyl isothiocyanates (BITC) and observed a 43% reduction in tumor growth. This study also showed a reduction in phosphorylation of phosphatidylinositide 3-kinase (PI3K), protein kinase B (AKT), pyruvate dehydrogenase kinase (PDK), forkhead box O3A (FOXO3A), FOXO1, and mammalian target of rapamycin (mTOR) in response to treatment with BITC. Phenyethyl isothiocyanates (PEITC) have been shown to reduce cancer growth by inhibiting AKT [56]. 

While studies involving moringa isothiocyanates are limited, several studies with other isothiocyanates, along with our preliminary studies in vitro with moringa isothiocyanates, suggest that this compound may open new frontiers in cancer therapeutics. 

### 6.1. Regulation of Cell Proliferation

Based on previous studies [52,57], *M. oleifera* has been confirmed to selectively inhibit the proliferation of different cell lines including lung cancer A549, human hepatocellular carcinoma HepG2, breast cancer MDA-MB-231, and colon cancer HCT-8 cells. Notably, the inhibitory rate of *M. oleifera* on the growth of neuroblastoma SH-SY5Y cells is up to 95%. In addition, *M. oleifera* leaf extract has been reported to have an anti-proliferative effect on KB cells, which is evaluated by cell morphologic change, cell viability, and internucleosomal DNA fragmentation [58].

### 6.2. Cell Cycle Arrest and Apoptosis

Apoptosis has been recognized to play an important role in maintaining cellular homeostasis through selective removal of damaged cells. The ability to induce apoptosis is a major mechanism of certain anti-tumor drugs. Previous studies have reported that isothiocyanates isolated from *M. oleifera* leaf extract are able to induce apoptosis in different cancer cells [58,59]. These studies have also reported that *M. oleifera* extract can inhibit the proliferation of cancer cells, but the molecular mechanisms are still limited. Various signaling pathways or associated mechanisms involved in apoptosis during the application of *M. oleifera* are highly correlated with the activation of caspase signaling. The extract from *M. oleifera* at different doses can lead to the increase in average sub-G1 populations during a 6 h administration in A549 lung cancer cells. Meanwhile, caspase-3 is downregulated and cleaved caspase-3 is upregulated upon the administration of *M. oleifera* leaf extract in a dose-dependent manner [60]. In addition, the administration of *M. oleifera* leaf extract resulted in a time-dependent increase of phosphor-c-Jun N-terminal kinase (p-JNK) and phosphor-extracellular signal-related kinase (p-ERK), without changes in total JNK or ERK protein, hinting at the possibility of a pro-apoptotic role of *M. oleifera* via activation of these kinases in human melanoma A2058 cells [61]. Interestingly, in cholangiocarcinoma (CCA), the phosphorylation levels of phospho-p44/42 MAPK (ERK1/2) and phospho-p38 MAPK increased in *M. oleifera* seed extract treated RMCCA1 cells, suggesting that the activity levels of anti- and pro-apoptotic signaling proteins may determine the apoptotic nature of this compound [62]. The extracts from *M. oleifera* leaves and bark also effectively arrest cell cycle progression at the G2/M phase and increase apoptosis in breast and colorectal cancer cell lines such as MDA-MB-231 and HCT-8 cells, which could be attributed to the bioactive compounds such as eugenol, isopropyl isothiocynate, D-allose, and hexadeconoic acid ethyl ester [52].

Additionally, the checkpoint failure of cell cycle usually causes genetic mutations and genomic rearrangements, thereby causing genetic instability as one of the major factors of cancer progression. Increasing evidence suggests that a variety of anti-cancer agents can induce cell cycle arrest at a certain checkpoint, thus inducing the apoptosis of cancer cells [63,64]. Jung has also found that cyclin D1 can be significantly downregulated in *M. oleifera* aqueous leaf extract-treated cells in a dose-dependent manner. Moreover, the treatment with *M. oleifera* leaf extract can induce an elevation in the sub-G1 cell population during cell cycle in a dose-dependent manner in human pancreatic cancer cell line (PANC-1 cells) and reduce the expression of p65, p-IkBα, and IkBα proteins [53], which further supports that *M. oleifera* leaf extract is a potential phytochemical to target cancer cells through arresting cell cycle. 

### 6.3. Synergistic Effect on Chemotherapeutic Drugs

Multi-drug resistance (MDR) is one of the major reasons for chemotherapeutic failure. MDR to chemotherapeutic drugs often leads to reduced treatment efficacy and cancer recurrence [65]. It is well known that phytochemical compounds have the advantages of low toxicity, weak side effects, multiple targets, and less tumor resistance as well as anti-tumor and immune-regulatory functions [65]. Therefore, natural compounds with reversed MDR have become the focus of anticancer studies. Although *M. oleifera* has not yet developed into a commercial chemopreventive agent, previous findings have revealed that the chemotherapeutic drug doxorubicin combined with *M. oleifera* callus and leaf extracts produces robust synergy on the growth inhibition of HeLa cells, which is also correlated with apoptotic induction [66]. The application of currently used anticancer drugs combined with *M. oleifera* could be a novel therapeutic strategy for cancers.

### 6.4. Regulating Enzyme Activity

A balance and the induction of Phase I and II drug metabolizing enzymes is a well-known defense against chemical carcinogens [67]. The loss of GSH and GST activity can be restored by *M. oleifera* pod extract, which offers a major protective role in carcinogenesis [67,68]. The hydro-alcoholic drumstick extract from *M. oleifera* as a bifunctional inducer can induce both Phase I and Phase II enzymes and improve the levels of hepatic cytochrome b5, cytochrome P450, and GST [69]. It is also reported that the antioxidant properties of *M. oleifera* is closely correlated with its potential as a chemo preventative agent. In addition, *M. oleifera* pod extract (200 and 400 mg/kg body weight; p.o.) and its isolated saponin (50 mg/kg body weight; p.o.) can attenuate 7,12-dimethylbenz[a]anthracene (DMBA)-induced renal carcinogenesis in mice through effectively suppressing renal oxidative stress and toxicity [70].

Based on the above comprehensive analysis from several angles, *M. oleifera* may exerts its anti-tumor effects by modulating multiple signaling pathways, including inducing cell apoptosis, triggering cell cycle arrest, inhibiting cell proliferation, suppressing angiogenesis and metastasis, enhancing drug metabolism, and synergizing with chemotherapeutic agents.

## 7. Modulation of Blood Glucose

Diabetes mellitus (DM) is a chronic metabolic disorder and the pharmacological actions of the leaves of *M. oleifera* have been reported for the traditional treatment of diabetes [71]. For example, *M. oleifera* has been shown to improve plasma glucose disposal in Goto-Kakizaki (GK) Wistar DM rats [72]. Similarly, the methanol extract from its fruit powder is rich in N-benzyl thiocarbamates, N-benzyl carbamates, and benzyl nitriles which can trigger the release of insulin from pancreatic beta cells of rodents, suppress cyclooxygenase activity, and inhibit lipid peroxidation [73]. *M. oleifera* has been found to significantly reduce glucose to normal levels without any obvious cytotoxicity when compared to the alloxan-induced type 2 diabetic rats from the model group [74]. The supplementation of the aqueous extract from *M. oleifera* leaves at the dose of 100 mg/kg can improve insulin sensitivity, increase total antioxidant capacity (TAC), and improve immune tolerance [75], which is consistent with another report that *M. oleifera* can ameliorate glucose intolerance [76]. *M. oleifera* extract can also reduce diabetes-related complications. Recent studies have shown that the administration of *M. oleifera* leaf extract for six weeks plays a critical role in reducing diabetic complications by protecting diabetes-induced renal damage and inflammation in a streptozotocin-induced diabetes rat model [77]. In addition, the administration of *M. oleifera* seed powder can ameliorate diabetic nephropathy and restore normal histology of both kidney and pancreas when compared with a diabetic positive control group [78]. 

## 8. Future Perspectives

Autophagy is an evolutionarily conserved process whereby cytoplasm and cellular organelles are degraded in lysosomes for amino acid and energy recycling, thus executing its cytoprotective role [79]. Basal autophagy plays a critical role in cellular homeostasis. Autophagy can be induced under different conditions such as nutrient deprivation, endoplasmic reticulum (ER)-stress, and exposure to anticancer drugs. However, defective or impaired autophagy has been implicated in the pathogenesis of diverse disease states, including microbial infection, inflammation, neuronal degeneration, aging, and cancer [80,81,82,83]. The induction or upregulation of autophagy appears to decrease the susceptibility to pro-apoptotic insults, which may have further benefits [84]. Recently, the functional status of autophagy during chronic disease processes has attracted increasing attention. Notably, the upregulation of autophagy mediated by a wide range of phytochemicals such as resveratrol, curcumin, and quercetin can exert anti-inflammatory, anti-tumor and anti-aging effects. More importantly, *M. oleifera* can be treated as a nutraceutial product or food because of its safety, which will motivate the exploration of its potential to activate autophagy for the prevention and treatment of chronic diseases in the future. 

## 9. Conclusions

*M. oleifera* possesses a wide range of medicinal and therapeutic properties through executing its potent anti-inflammatory activity, inhibiting the activation of NF-κB and PI3K/Akt pathways, mitigating oxidative stress by scavenging free radicals, and enhancing neuroprotective roles. In addition, *M. oleifera* can reduce the risk of cancer and modulate blood glucose, although the underlying mechanisms remain to be further explored. Therefore, *M. oleifera* provides the potential for the prevention or treatment of a series of chronic diseases.
